# Vismodegib treatment in locally advanced basal cell carcinoma limited to the facial region: a single-center experience

**DOI:** 10.1186/s12885-025-14914-2

**Published:** 2025-10-06

**Authors:** İvo Gökmen, Erdem Şen

**Affiliations:** Department of Medical Oncology, Mehmet Akif Ersoy State Hospital, Çanakkale, Türkiye

**Keywords:** Adverse events, Basal cell carcinoma, Prognosis, Therapy, Vismodegib

## Abstract

**Introduction:**

Basal cell carcinoma (BCC) is the most common non-melanoma skin cancer. Treatment typically begins with surgical intervention. However, in cases of locally advanced and metastatic BCC (laBCC and mBCC), Hedgehog signaling pathway inhibitors such as sonidegib and vismodegib are used.

**Materials and methods:**

This retrospective study included 28 adult patients with laBCC who were treated with 150 mg daily oral vismodegib at Mehmet Akif Ersoy State Hospital between 2018 and 2023. Patients were monitored until disease progression, intolerable side effects, or treatment discontinuation. Treatment efficacy was evaluated using objective response rate (ORR) and disease control rate (DCR). Safety was assessed based on adverse events (AEs) reported in accordance with the CTCAE v. 5.0 classification. Statistical analyses, including overall survival (OS) and progression-free survival (PFS), were performed and analyzed using SPSS version 25.

**Results:**

The overall ORR was 89.3%, with 39.3% of patients achieving complete response (CR). The median PFS was 15.1 months, and the median OS was 37.5 months. Female gender and prior surgical interventions were identified as independent prognostic factors for PFS, while ECOG-PS score and the duration of vismodegib exposure were significant predictors of OS. AEs were reported in 78.6% of patients, with dysgeusia and muscle spasms being the most prevalent. Grade 3 or 4 toxicity occurred in 14.5% of patients, and 13% (*n* = 3) discontinued treatment due to AEs. Patients receiving treatment for ≥ 12 months experienced a higher incidence of AEs (92.9%) compared to those treated for < 12 months (64.3%).

**Conclusion:**

Vismodegib demonstrated high efficacy in treating patients with laBCC, with a significant proportion achieving CR. However, long-term treatment was associated with an increased incidence of AEs, highlighting the need for careful monitoring of safety in prolonged therapies.

## Introduction

Basal cell carcinoma (BCC) constitutes 85% of non-melanoma skin cancers and is the most commonly diagnosed malignancy worldwide. Although precise incidence and mortality rates are unclear due to numerous unreported cases [1], GLOBOCAN 2020 data indicate approximately 8 million new BCC cases [2]. BCC is primarily localized to the head and neck region, predominantly affecting elderly individuals. It has a low metastatic potential; however, due to its slow growth, the average delay in diagnosis can extend up to 25 months. Such delays may result in the progression of the disease to the locally advanced BCC (laBCC) stage [3].

The treatment of laBCC is complex, and a standardized approach has not yet been established [4]. Non-surgical options, such as imiquimod, photodynamic therapy, and 5-fluorouracil, are available for small and superficial lesions; however, these methods are inadequate for advanced tumors and are associated with disadvantages such as high recurrence rates [5]. Surgical excision is preferred as the primary treatment for small lesions, with the goal of complete tumor removal using wide excision margins. Larger lesions may require reconstructive surgery. However, surgery may not always be feasible in laBCC due to the risk of functional impairment and cosmetic deformity [6]. In such cases, radiotherapy may be considered as an alternative, but it poses risks such as limited disease control, healing difficulties, and the development of secondary tumors [7]. For cases where surgery and radiotherapy are insufficient, particularly in laBCC, there is a need for more advanced and targeted therapies.

The central mechanism underlying the development of BCC is the overactivation of the Hedgehog (Hh) signaling pathway. This pathway is typically disrupted by inhibition of the patched homolog 1 (PTCH1) gene or, less frequently, by activation of the smoothened homolog (SMO) protein. In normal adult cells, the Hh signaling pathway remains inactive, but mutations in these components lead to abnormal activation, resulting in uncontrolled cell proliferation and tumor growth— a process observed in the majority of sporadic BCC cases [8]. These molecular mechanisms provide the foundation for current treatment strategies. The introduction of Hh pathway inhibitors represents a significant advancement in the treatment of laBCC. Currently, the U.S. Food and Drug Administration (FDA) and the European Medicines Agency (EMA) have approved two SMO inhibitors for the treatment of laBCC and metastatic BCC that are unsuitable for surgery or radiotherapy, or that have recurred following treatment [9].

Vismodegib is the first molecule approved among Hh pathway inhibitors [10]. While the efficacy and safety of vismodegib have been demonstrated in controlled clinical trials, a more comprehensive assessment of this treatment can be achieved through the evaluation of real-world data [9, 10]. In this study, the effectiveness and safety of vismodegib in the treatment of laBCC will be assessed in a real-world setting. Such data provide valuable insights into the challenges encountered during treatment and the outcomes achieved, contributing to the optimization of clinical practice.

## Materials and methods

This retrospective study was conducted at the Medical Oncology Department of Mehmet Akif Ersoy State Hospital, a secondary healthcare institution in Çanakkale, Türkiye. The study included 28 adult patients diagnosed with laBCC between January 2018 and December 2023. Eligible patients had histologically confirmed laBCC and were deemed unsuitable for surgery or radiotherapy due to medical contraindications or experienced disease recurrence following two or more surgical interventions in the same region. Additionally, patients for whom curative resection was either not feasible or likely to result in significant morbidity or deformity were included. All patients received a daily dose of 150 mg oral vismodegib (Erivedge^®^) and were monitored until disease progression, intolerable side effects, or treatment discontinuation.It should be noted that sonidegib, although approved in several countries for the treatment of laBCC, is not reimbursed by the national health system in Türkiye and was not accessible to patients during the study period. Therefore, all patients in this cohort were treated exclusively with vismodegib.

After the initial treatment, all patients were re-evaluated at four weeks and subsequently monitored at intervals of up to eight weeks. At each follow-up, laboratory tests were conducted, and lesions were photographed with a visible scale for documentation. CT/MRI imaging was performed when necessary, and adverse events were recorded. Treatment efficacy was evaluated by reviewing medical records and clinical photographs, measuring the total tumor diameter, the largest lesion’s diameter, and the number of lesions. Partial response (PR) was defined as a ≥ 30% reduction in visible or radiographic tumor size or healing of ulceration, while complete response (CR) was defined as the complete clinical disappearance of the tumor.

The primary objective of this study was to evaluate the effectiveness of vismodegib in terms of progression-free survival (PFS) in patients with laBCC. Secondary endpoints included overall survival (OS), treatment efficacy, and treatment safety.To further assess treatment efficacy, objective response rate (ORR) and disease control rate (DCR) were calculated. ORR was defined as the sum of CR and PR, while DCR included CR, PR, and stable disease (SD). PFS was defined as the time from treatment initiation to disease progression or death, and OS was defined as the time from treatment initiation to death or the last recorded follow-up. Safety assessments were based on adverse events (AEs) reported according to CTCAE v. 5.0 classification and severity grading criteria.

### Statistical analysis

All statistical analyses were conducted using the Statistical Package for the Social Sciences (SPSS) version 25 (IBM Corp., Armonk, NY). Categorical variables were reported as frequencies and percentages, while continuous variables were presented as medians (minimum-maximum). OS and PFS were analyzed using the Kaplan-Meier method, with group comparisons performed using the log-rank test. Univariate analyses for both OS and PFS were conducted using the Cox regression model. A p-value of < 0.05 was considered statistically significant, while p-values < 0.1 were considered to approach significance and were included in the analyses.

## Results

### Patient demographics and clinical characteristics

A total of 28 patients participated in this study, of whom 74% (*n* = 21) were female. The majority of patients were elderly, with a median age of 80.7 years (range: 59–92), and 35.7% (*n* = 10) were over 85 years of age. The median age was 79.2 years (range: 62–92) for females and 87.6 years (range: 59–92) for males. At the initiation of treatment, 82.1% (*n* = 23) of the patients had at least one comorbidity. Regarding performance status, 67.9% (*n* = 19) of the patients were assessed as ECOG-PS 0–1, and 32.1% (*n* = 9) as ECOG-PS ≥ 2. All lesions were localized to the facial region. A single lesion was observed in 67.9% (*n* = 19) of the patients, whereas 32.1% (*n* = 9) had multiple lesions. The most frequently affected areas were the nose and cheeks, which were involved in 32.1% (*n* = 9) of cases, followed by the forehead (28.6%, *n* = 8) and the periorbital region (21.4%, *n* = 6). The median size of the lesions was 4 cm (SD: 2.2 cm; range: 1.5–10 cm). Prior to Vismodegib treatment, 53.6% (*n* = 15) of the patients had undergone multiple surgical interventions, and 32.1% (*n* = 9) had received radiotherapy (see Table 1 for patient characteristics).

**Table 1:** Summary of patients’ demographic and clinical characteristics

Patients Characteristics	*n*, %
Age (years) Mean (± SD) Median (range)	79.7 (± 9.5) 80.7 (59–92)
Age Group (years) < 85 ≥ 85	18, 64.3 10, 35.7
Gender Female Male	21, 75 7, 25
Comorbidity Status Yes No	23, 82.1 7, 17.9
ECOG PS 0–1 ≥ 2	19, 67.9 9, 32.1
Number of Lesions 1, ≥ 2	19, 67.9 9, 32.1
Primary Tumor Location Eyes Nose Lips Ears Cheek Forehead Temple Chin Scalp	6, 21.4 9, 32.1 2, 7.1 2, 7.1 9, 32.1 8, 28.6 1, 3.6 3, 10.7 1, 3.6
Previous Surgery Yes No	15, 53.6 13, 53.6
Previous Radiotherapy Yes No	9, 32.1 19, 67.9

*Abbreviations*: *SD* standard deviation, *ECOG PS* Eastern Cooperative Oncology Group Performance Status, *n* number

## Treatment processes and responses

In the study group, the median follow-up duration after the initiation of vismodegib treatment was calculated as 27 months (range: 5.7–77.9 months), while the median treatment duration was determined as 12 months (range: 2–38 months). As of the data cutoff date, 17.9% of the patients (*n* = 5) were still receiving treatment. Treatment was discontinued in 82.1% of the patients (*n* = 23), with the most common reasons for discontinuation being patient preference (30.4%, *n* = 7), physician decision (26.1%, *n* = 6), and death (21.7%, *n* = 5). Less frequent reasons for discontinuation included adverse events (13.2%, *n* = 3) and disease progression (8.7%, *n* = 2).

The overall ORR was 89.3% (*n* = 25), with CR observed in 39.3% (*n* = 11) and PR observed in 50.0% (*n* = 14) of patients. The DCR was calculated as 92.9% (*n* = 26). In the analysis of treatment response, the median time to response initiation was 4 weeks (range: 3–6 weeks), and the median time to achieve the best response was 3.7 months (range: 1.2–12 months) (for further details on treatment outcomes, refer to Table 2).

**Table 2:** Treatment results of the study population

		*n*, %
Best Response	CR	11, 39.3
	PR	14, 50
	ORR	25, 89.3
	SD	1, 3.6
	PD	2, 7.1
	DCR	26, 92.9
Time to Best Response	Month, mean (± SD) Median (range)	4.9 (± 3.6) 3.7 (1.2–12.8)
Exposure to Vismodegib	Month, mean (± SD) Median (range)	13.4 (± 8.6) 12.0 (2.0–38.0)
	< 12 months	14, 50.0
	≥ 12 months	14. 50.0
Reasons for Treatment	AEs	3, 13.0
Discontinuation	Physician decision	6, 26.1
	Patient dicision	7, 30.4
	Disease progression	2, 8.7
	Death	5, 21.7
Median Follow-Up Time	Month, mean (± SD) Median (range)	29.8 (± 18.9) 27.0 (5.7–77.9)

*Abbreviations*: *CR* complete response, *PR* partial response, *ORR* objective response rate, *SD* stable disease, *PD* progressive disease, *DCR* disease control rate, *AEs* adverse events, *SD* standard deviation

## Survival analyses

**Fig. 1:**
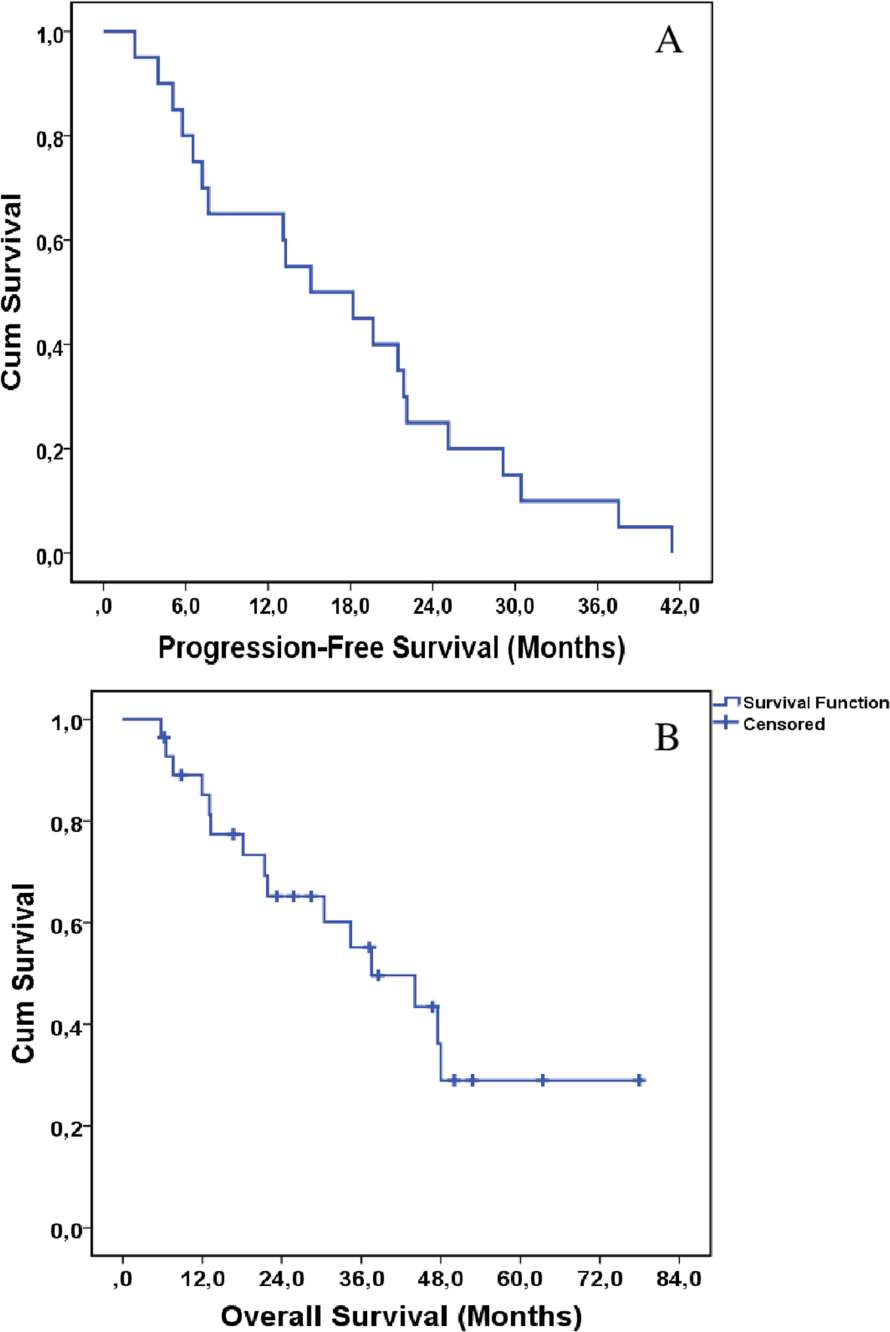
Shows Kaplan-Meier curves for PFS (**A**) and OS (**B**) in the entire study group

Disease progression was observed in 71.4% of the patients (*n* = 20). The median PFS was calculated to be 15.1 months, with a 95% confidence interval (CI) ranging from 4.3 to 25.9 months (see Fig. 1A). The PFS rates at one and two years were determined to be 65% and 25%, respectively.As of the data cutoff date, 53.6% of the patients (*n* = 15) had succumbed. The median OS was found to be 37.5 months (95% CI: 20.1–54.8) (see Fig. 1B). The OS rates at one and two years were calculated to be 78.5% and 39.2%, respectively.

### Analysis of prognostic factors

In the univariate analysis of factors potentially impacting PFS, statistically significant differences were observed for gender (*p* < 0.05) and previous surgical interventions (*p* < 0.1). The results of the multivariate analysis confirmed that female gender (*p* = 0.016, HR: 3.9, 95% CI: 1.3–12.1) and previous surgical interventions (*p* = 0.028, HR: 3.3, 95% CI: 1.1–9.7) are independent risk factors associated with PFS (see Table 3).

**Table 3:** Univariate and multivariate analysis of PFS-Associated factors in LaBCC patients treated with vismodegib

Factor	Median PFS		Univariate Analysis		Multivariate Analysis		
	(95%Cl), months	*p (log-rank)*	HR	*p-value*	HR	95%Cl	*p-value *
Age Group							
< 85 ≥ 85	18.2 (9.2–27.2) 13.8 (NA-29.7)	0.52	1.353	0.52	-		
Gender							
Female Male	19.6 (13.6–25.6) 7.2 (4.9–9.4)	0.04	2.886	0.05	3.956	1.291–12.211	0.016
Comorbidity Status							
Yes No	13.2 (9.3–17.2) 21.4 (NA-47.7)	0.28	2.201	0.29	**-**		
ECOG PS							
0–1 ≥ 2	18.2 (9.8–26) 6.5 (4.8–8.2)	0.52	1.399	0.59	**-**		
Number of Lessions							
1 ≥2	19.6 (9.3–29.9) 13.3 (NA-32.6)	0.29	1.811	0.22	**-**		
Previous Surgery							
Yes No	12.3 (12.8–13.5) 21.9 (6.3–37.4)	0.07	2.495	0.08	3.321	1.136–9.710	0.028
Previous Radiotherapy							
Yes No	13.1 (2.2–24.1) 18.2 (2.1–32.8)	0.12	2.154	0.14	**-**		

*Abbreviations*: *PFS* progression-free survival, *HR* hazard ratio, *CI* confidence interval, *ECOG PS* Eastern Cooperative Oncology Group Performance Status

In the univariate analysis of factors that may influence OS, statistically significant differences were found for age (*p* < 0.1), ECOG-PS score (*p* < 0.05), and duration of exposure to vismodegib (*p* < 0.05). The multivariate analysis corroborated that the ECOG-PS score (*p* = 0.07, HR: 2.075, 95% CI: 0.908–8.054) and duration of exposure to vismodegib (*p* = 0.04, HR: 3.708, 95% CI: 1.033–13.310) are independent prognostic factors associated with OS. However, given that the p-value for ECOG-PS is close to the significance threshold, these results should be interpreted with caution (see Table 4).

**Table 4:** Univariate and multivariate analysis of OS-Associated factors in LaBCC patients treated with vismodegib

Factor	Median OS		Univariate Analysis		Multivariate Analysis		
	(95%Cl), months	*p* (log-rank)	HR	*p*-value	HR	95%Cl	*p*-value
Age Group							
< 85 ≥ 85	47.5 (NA-NA)	0.06	2.675	0.06	2.082	0.725–5.979	0.17
Gender							
Female Male	44.1 (26.5–61.7) 34.4 (0.0-77.6)	0.63	1.320	0.63	-		
Comorbidity Status							
Yes No	34.4 (22.1–46.5) 48.0 (25.9–70.0)	0.52	1.519	0.52	-		
ECOG PS							
0–1 ≥ 2	47.5 (31.7–63.2) 21.9 (11.3–32.4)	0.03	3.663	0.04	2.705	0.908–8.054	0.07
Number of Lessions							
1 ≥2	44.1 (30.2–57.9) 30.4 (7.6–53.2)	0.93	1.051	0.93	-		
Previous Surgery							
Yes No	NA (NA-NA) 34.5 (17.1–51.5)	0.11	2.385	0.12	-		
Previous Radiotherapy							
Yes No	NA (NA-NA) 37.5 (14.9–60.1)	0.14	2.395	0.15	-		
Exposure to Vismodegib							
< 12 months ≥ 12 months	21.4 (9.3–33.6) 47.5 (36.6–55.2	0.02	3.152	0.03	3.708	1.033–13.310	0.04

*Abbreviations*: *OS* overall survival, *HR* hazard ratio, *CI* confidence interval, *ECOG PS *Eastern Cooperative Oncology Group Performance Status, *NA* not available

## Adverse events

Adverse events associated with vismodegib treatment were observed in 78.6% of the patients (*n* = 22). Multiple AEs were reported in 64.3% of the patients (*n* = 18). The most common adverse events included dysgeusia, reported in 67.9% of patients (*n* = 21), followed by muscle spasms in 57.1% (*n* = 16) and alopecia. Other notable AEs included decreased appetite (37.5%, *n* = 10) and fatigue (28.6%, *n* = 8).

The majority of adverse events were of mild severity, with 49.5% classified as Grade 1, while 14.5% were categorized as Grade 3 or 4 toxicity. Alopecia, when reported, was most frequently classified as a Grade 4 adverse event (75%). Rarely, AEs (13%, *n* = 3) were the reason for treatment discontinuation (see Table 2). The safety profile of AEs is detailed in Table 5, which presents a comparative analysis of the findings from this study with results from the ERIVANCE and STEVIE studies.

**Table 5:** Adverse event profile of vismodegib in labcc: A comparison of the present study with ERIVANCE and STEVIE trials

	Present Study (*n* = 28)	STEVİE (*n* = 1215)	ERİVANCE (*n* = 104)
**TEAEs**			
Yes	22, 78.6	1192, 100	104, 100
**Any grade, n, %**			
Dysgeusia	21, 67.9	663, 54.6	58, 55.8
Muscle spasm	16, 57.1	807, 66.4	74, 71.2
Alopecia	16, 57.1	747, 61.5	69, 66.3
Decreased appetite	10, 37.5	303, 24.9	29, 27.9
Fatigue	8, 28.6	201, 16.5	45, 43.3
Decreased weight	7, 25.0	493, 40.6	54, 51.9
Arthralgia	5, 17.9	124, 10.2	17. 16.3
Pruritus	4, 14.3	-	11, 10.6
Abdominal spasms	3, 10.7	-	-
Diarrhea	3, 10.7	197, 16.2	28, 26.9
Vemoting	3, 10.7	102, 8.4	18, 17.3
Consipation	2, 7.1	116, 9.5	20, 19.2

*Abbreviations*: *TEAEs* treatment-emergent adverse events, *n* number

## Subgroup analysis according to duration of vismodegib exposure

In the analysis based on the duration of vismodegib treatment, a lower median age was observed in the long-term treatment group (≥ 12 months) compared to the short-term group (78 years vs. 83 years; *p* = 0.066), although this difference did not reach statistical significance. In this group, the number of patients with ECOG-PS scores ≥ 1 was significantly lower (2 patients vs. 7 patients; *p* = 0.043), and the CR rate was notably higher (9 patients vs. 2 patients; *p* = 0.018).

**Fig. 2:**
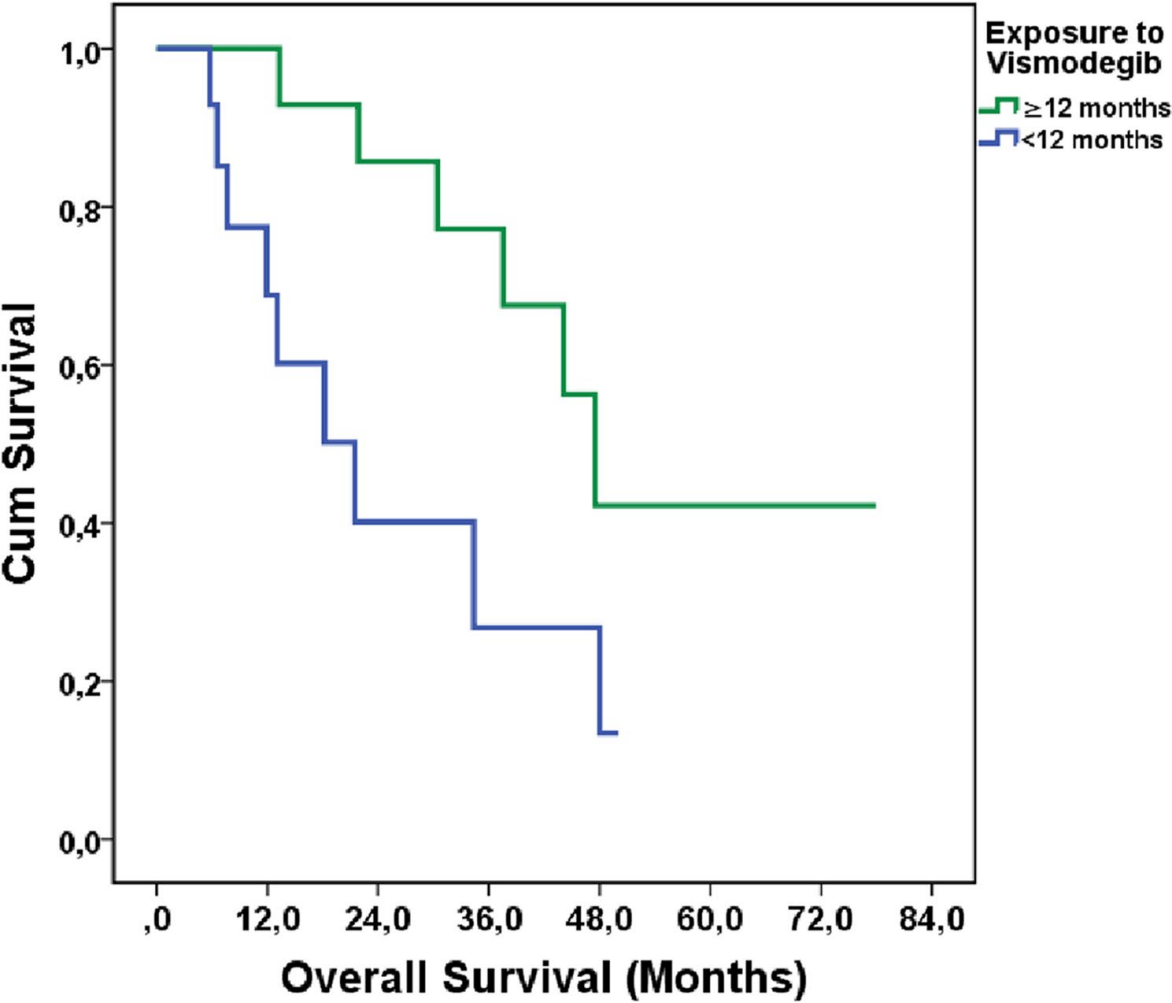
Kaplan-Meier Analysis of OS Based on Vismodegib Exposure Duration

Regarding survival outcomes, the median PFS for patients receiving treatment for ≥ 12 months was 21.8 months, while the OS was 47.5 months. In contrast, patients treated for < 12 months had a median PFS of 7.7 months and an OS of 21.5 months (*p* = 0.02). The OS data were visualized using Kaplan-Meier curves **(**see Fig. 2**)**. Additionally, concerning (AEs, AEs were observed in 64.3% of patients treated for < 12 months, compared to 92.9% in those treated for ≥ 12 months.

## Discussion

Vismodegib is a synthetic small molecule that inhibits the SMO protein, a key component of the Hh signaling pathway. Positive outcomes from early-phase clinical trials [11, 12] led to its FDA approval in 2012. It is particularly favored in patients for whom surgery and radiotherapy are unsuitable or pose significant cosmetic and functional risks [13, 14]. Two major international studies, ERIVANCE and STEVIE, demonstrated clinical response rates of 60.3% and 68.5% in laBCC, respectively [9, 10]. Following this success, more comprehensive evaluations of the efficacy and safety of vismodegib have been conducted. Although real-world data remain limited, meta-analyses and systematic reviews continue to support vismodegib as a reliable and effective treatment option [13, 15–26]. A meta-analysis encompassing 16 studies reported an overall response rate of 69% and a complete response rate of 31% [15].

According to our data, the clinical response rate was 89.3%. Similar findings were reported in a study conducted in Turkey [20]. The higher rates observed compared to previous clinical trials may be attributed to the small sample size, flexible clinical criteria, and the absence of multiple biopsies for histological confirmation. However, in the prospective RegiSONIC study conducted in the United States with laBCC patients, a clinical response rate of 85.1% was reported [27], while studies from Greece and Sweden reported clinical response rates exceeding 90% [25, 26].

The median age of our cohort (81 years) was higher than that reported in the ERIVANCE (62 years) and STEVIE (72 years) studies [9, 10]. It is important to consider that age may influence clinical outcomes, as older patients tend to present with more advanced disease or multiple lesions due to prolonged exposure to ultraviolet radiation [28].

In our study, the median time to response for vismodegib was 4 weeks, which is significantly shorter compared to the ERIVANCE (4.6 months) and STEVIE (3.7 months) studies [9, 10]. Maximum response was achieved in a median of 3.7 months, which is also shorter than in previous studies. The rapid response observed in real-world clinical practice may be attributed to differences in clinical settings, further supporting the effectiveness of our treatment.

The median treatment duration was calculated as 12 months, which is nearly identical to the median duration of 12.7 months reported in the laBCC cohort of the ERIVANCE trial [10]. The median PFS in our study was 15 months, which is comparable to the 12.9 months reported for laBCC patients in the ERIVANCE study but shorter than the 23.2 months reported in the STEVIE trial [9, 10]. Although the reported progression rate was 71.4%, only two patients experienced true clinical or radiological tumor progression during vismodegib therapy, indicating primary resistance. In these cases, vismodegib had been administered for at least 2.5 months prior to progression, allowing sufficient time to assess treatment response. Additionally, five patients who had previously achieved either a CR or a PR died from causes unrelated to laBCC, such as advanced age or comorbidities, and were therefore classified as progression events according to the predefined PFS criteria. Furthermore, some patients who had discontinued vismodegib after achieving a treatment response experienced disease progression during a drug-free follow-up period. These cases reflect the natural course of the disease after treatment cessation rather than resistance that developed during active therapy. Among patients who were followed without disease progression after treatment discontinuation, the mean disease-free duration was 12.18 months (range: 3.09–21.52 months) in those with CR and 10.27 months (range: 1.74–17.28 months) in those with PR. These findings suggest that the treatment responses were not short-lived and were followed by clinically meaningful remission periods. Therefore, the high ORR observed in our study reflects a durable clinical benefit, and the reported progression rate should be interpreted with caution in this context. In addition to PFS, OS outcomes were also evaluated. The median OS was 37.5 months, with 1-year and 2-year OS rates of 78.5% and 39.2%, respectively. These OS rates are lower than the 1-year and 2-year rates of 93.2% and 85.5% reported in the ERIVANCE study [9]. This discrepancy may be related to the limited number of patients with long-term follow-up in this cohort.

The study by Słowinska M et al. examined prognostic factors for PFS and OS, emphasizing the importance of gender and treatment response. The study demonstrated that female gender was an independent positive factor for PFS, and a CR or partial treatment response significantly improved OS [29]. Our findings are consistent with these results; in our data, female gender was found to have a positive impact on PFS, and previous surgical interventions were identified as independent factors positively influencing PFS. In our OS analyses, a good ECOG-PS score and long-term vismodegib treatment were identified as independent prognostic factors for OS. Notably, the advanced age of patients did not appear to compromise treatment efficacy. This observation is corroborated by the subgroup analyses by age in the ERIVANCE and STEVIE clinical trials, which reported similar efficacy across different age groups [9, 10].

Despite the high clinical activity of vismodegib, its major limitation is the high incidence of adverse events; in the ERIVANCE and STEVIE studies, adverse events were reported in 98–100% of patients [9, 10]. Common adverse events included alopecia, muscle spasms, nausea, dysgeusia, decreased appetite, weight loss, and fatigue, with grade 3 or 4 events reported at lower frequencies.

Vismodegib treatment showed a relatively favorable tolerability profile. At least one adverse event was reported in 78.6% of patients, with the most common being dysgeusia (67.9%), muscle spasms (57.1%), and alopecia (57.1%). While it is essential to consider adverse events, the majority were mild (Grade 1–2) in severity. In our study, treatment was discontinued due to adverse events in only 13% of patients. In comparison, the discontinuation rates due to adverse events were reported as 21.2% in the ERIVANCE study and 28.7% in the STEVIE study [9, 10]. The retrospective design of our study may have contributed to the underreporting of subjective adverse events, particularly those self-reported by patients. Additionally, the lower incidence of certain adverse events may be associated with more stringent patient selection criteria or improved management of side effects.

Considering these side effects, discontinuing vismodegib treatment and adopting a follow-up strategy, especially in patients who have achieved a complete response, may be an effective option to reduce toxicity and improve treatment tolerability. The study by Herms et al. supports this approach; among patients who discontinued vismodegib and experienced recurrence, 85% achieved an objective response upon reinitiation of treatment, and 42% became eligible for surgery [30]. Furthermore, the MIKIE study compared two different intermittent regimens with eight-week intervals and demonstrated that treatment discontinuation did not adversely affect the efficacy of vismodegib. This study also showed that intermittent dosing was associated with fewer grade ≥ 3 adverse events (AEs) compared to the STEVIE study, suggesting that this strategy may help mitigate adverse events [31]. In cases where vismodegib is not tolerated or treatment is discontinued, radiation therapy may serve as a salvage option. However, its use is often limited in elderly patients or in facial lesions due to toxicity concerns and anatomical constraints [32].

In addition, considering the high objective response rates and rapid response time observed in our study, the use of vismodegib as a neoadjuvant or cytoreductive therapy prior to surgery may also be considered. This approach could be particularly beneficial in anatomically challenging areas where surgical resection carries a high risk of morbidity or may compromise important cosmetic and functional structures. A recent case report demonstrated successful neoadjuvant treatment with sonidegib, resulting in improved surgical eligibility [33]. Although this strategy was not applied in our study, our findings may provide a basis for future prospective studies evaluating the use of vismodegib for this purpose.

Furthermore, patients receiving vismodegib should be monitored for the development of new skin lesions, including cutaneous squamous cell carcinoma (SCC). The relationship between vismodegib use and the occurrence of cutaneous SCC remains inconclusive in the literature; while some studies have reported an increased incidence [34]others have found no such association [35]. No cases of cutaneous SCC were observed during the treatment period, indicating a favorable overall safety profile for vismodegib.

One of the primary limitations of vismodegib treatment is the development of resistance in BCC, which may shorten the duration of treatment response. The rate of secondary resistance has been reported in the literature to be approximately 20% [36], typically emerging after an average of 56 weeks [37]. However, resistance could not be conclusively evaluated in this cohort due to the retrospective design and heterogeneous reasons for treatment discontinuation.

The high mutation burden of BCC enhances its sensitivity to immunotherapy. For patients who develop resistance to or cannot tolerate vismodegib, the PD-1 inhibitor cemiplimab offers an effective alternative treatment option. In a multicenter, open-label phase II trial (Study 1620), cemiplimab was evaluated in patients with laBCC and mBCC who had progressed on or were intolerant to HHI therapy. The study reported an ORR of 31%, with responses observed across multiple anatomical sites and histological subtypes. Notably, 79% of responders achieved durable responses lasting ≥ 6 months, and the median duration of response had not been reached at the time of analysis. The treatment was generally well tolerated, with most adverse events being grade 1 or 2, and only a small proportion leading to treatment discontinuation [38, 39].Additionally, a recent case report described sustained clinical benefit even after discontinuation of cemiplimab, raising the possibility of immune memory contributing to long-term disease control. These findings highlight cemiplimab as a promising systemic option in the post-HHI setting, particularly for patients with limited surgical or radiotherapeutic alternatives [40].​ This highlights the need to improve accessibility and integrate PD-1 inhibitors into treatment algorithms, especially in resource-limited settings.

The primary limitations of this study are its retrospective and single-center design, which may lead to data deficiencies and underreporting of adverse events. Additionally, the small sample size limits the generalizability of the findings. The relatively short follow-up period in some patients has also resulted in limited long-term OS data. Moreover, since access to PD-1 inhibitors or alternative HHI agents is restricted in our country, none of the patients received these treatments, and post-progression therapeutic strategies could not be systematically evaluated. Furthermore, despite achieving favorable clinical responses, some patients voluntarily discontinued vismodegib and declined to restart treatment following disease progression. This limits the ability to evaluate the efficacy of re-treatment with the same agent; however, it also reflects the real-world challenges of maintaining treatment adherence in elderly populations.

## Conclusions

In conclusion, vismodegib appears to be a highly effective and well-tolerated treatment option for patients with laBCC, even in elderly populations with significant comorbidities. Our real-world findings demonstrate a high rate of durable clinical responses, with meaningful disease-free intervals following treatment discontinuation, particularly in those achieving a complete or partial response. Although the overall incidence of adverse events was high, the relatively low rate of severe toxicity and treatment discontinuation supports the feasibility of vismodegib in routine clinical practice. Importantly, true clinical or radiological progression during active treatment was observed in only a small proportion of patients, suggesting that many progression events captured by PFS metrics may not reflect true therapeutic failure. Future studies should further explore intermittent dosing strategies and the potential for re-treatment upon recurrence, as these may optimize long-term outcomes while minimizing cumulative toxicity. Moreover, expanding access to alternative therapies such as PD-1 inhibitors will be essential in managing patients who are resistant to or intolerant of Hh inhibitors.

## Data Availability

Data may be shared by the corresponding author upon reasonable request and subject to obtaining the necessary permissions.

## References

[1] Verkouteren JAC, Ramdas KHR, Wakkee M, Nijsten T. Epidemiology of basal cell carcinoma: scholarly review. Br J Dermatol. 2017;177(2):359–72. 28220485 10.1111/bjd.15321

[2] Ciążyńska M, Kamińska-Winciorek G, Lange D, Lewandowski B, Reich A, Sławińska M, et al. The incidence and clinical analysis of non-melanoma skin cancer. Sci Rep. 2021;11(1):4337. 33619293 10.1038/s41598-021-83502-8 PMC7900109

[3] Hoorens I, Vossaert K, Ongenae K, Brochez L. Is early detection of basal cell carcinoma worthwhile? Systematic review based on the WHO criteria for screening. Br J Dermatol. 2016;174(6):1258–65. 26872563 10.1111/bjd.14477

[4] Walling HW, Fosko SW, Geraminejad PA, Whitaker DC, Arpey CJ. Aggressive basal cell carcinoma: presentation, pathogenesis, and management. Cancer Metastasis Rev. 2004;23(3–4):389–402. 15197337 10.1023/B:CANC.0000031775.04618.30

[5] Foley P. Current landscape for treatment of advanced basal cell carcinoma. Australas J Dermatol. 2015;56(Suppl 1):1–7. 25715811 10.1111/ajd.12319

[6] Goldenberg G, Karagiannis T, Palmer JB, Lotya J, O’Neill C, Kisa R, et al. Incidence and prevalence of basal cell carcinoma (BCC) and locally advanced BCC (LABCC) in a large commercially insured population in the united states: A retrospective cohort study. J Am Acad Dermatol. 2016;75(5):957–e662. 27473450 10.1016/j.jaad.2016.06.020

[7] Likhacheva A, Awan M, Barker CA, Bhatnagar A, Bradfield L, Brady MS, et al. Definitive and postoperative radiation therapy for basal and squamous cell cancers of the skin: executive summary of an American society for radiation oncology clinical practice guideline. Pract Radiat Oncol. 2020;10(1):8–20. 31831330 10.1016/j.prro.2019.10.014

[8] Chmiel P, Kłosińska M, Forma A, Pelc Z, Gęca K, Skórzewska M. Novel approaches in non-melanoma skin cancers—a focus on Hedgehog pathway in basal cell carcinoma (BCC). Cells. 2022;11(20):3210. 10.3390/cells11203210 PMC9601130 36291078

[9] Basset-Séguin N, Hauschild A, Kunstfeld R, Grob J, Dréno B, Mortier L, et al. Vismodegib in patients with advanced basal cell carcinoma: primary analysis of STEVIE, an international, open-label trial. Eur J Cancer. 2017;86:334–48. 29073584 10.1016/j.ejca.2017.08.022

[10] Sekulic A, Migden MR, Basset-Seguin N, Garbe C, Gesierich A, Lao CD, et al. Long-term safety and efficacy of vismodegib in patients with advanced basal cell carcinoma: final update of the pivotal ERIVANCE BCC study. BMC Cancer. 2017;17(1):332. 28511673 10.1186/s12885-017-3286-5 PMC5433030

[11] Amin SH, Tibes R, Kim JE, Hybarger CP. Hedgehog antagonist GDC-0449 is effective in the treatment of advanced basal cell carcinoma. Laryngoscope. 2010;120(12):2456–9. 20927781 10.1002/lary.21145

[12] Von Hoff DD, LoRusso PM, Rudin CM, Reddy JC, Yauch RL, Tibes R, et al. Inhibition of the Hedgehog pathway in advanced basal-cell carcinoma. N Engl J Med. 2009;361(12):1164–72. 19726763 10.1056/NEJMoa0905360

[13] Peris K, Fargnoli MC, Garbe C, Kaufmann R, Bastholt L, Seguin NB, et al. Diagnosis and treatment of basal cell carcinoma: European consensus-based interdisciplinary guidelines. Eur J Cancer. 2019;118:10–34. 31288208 10.1016/j.ejca.2019.06.003

[14] Axelson M, Liu K, Jiang X, He K, Wang J, Zhao H, et al. U.S. Food and drug administration approval: vismodegib for recurrent, locally advanced, or metastatic basal cell carcinoma. Clin Cancer Res. 2013;19(9):2289–93. 23515405 10.1158/1078-0432.CCR-12-1956

[15] Xie P, Lefrançois P. Efficacy, safety, and comparison of Sonic Hedgehog inhibitors in basal cell carcinomas: A systematic review and meta-analysis. J Am Acad Dermatol. 2018;79(6):1089–e10017. 30003981 10.1016/j.jaad.2018.07.004

[16] Migden MR, Chang ALS, Dirix L, Stratigos AJ, Lear JT. Emerging trends in the treatment of advanced basal cell carcinoma. Cancer Treat Rev. 2018;64:1–10. 29407368 10.1016/j.ctrv.2017.12.009

[17] Cozzani R, Del Aguila R, Carrizo M, Sanchez S, Gonzalez A. Efficacy and safety profile of vismodegib in a real-world setting cohort of patients with advanced basal cell carcinoma in Argentina. Int J Dermatol. 2020;59(5):627–32. 32034775 10.1111/ijd.14780

[18] Jacobsen AA, Aldahan AS, Hughes OB, Shah VV, Strasswimmer J. Hedgehog pathway inhibitor therapy for locally advanced and metastatic basal cell carcinoma: A systematic review and pooled analysis of interventional studies. JAMA Dermatol. 2016;152(7):816–24. 27096888 10.1001/jamadermatol.2016.0780

[19] Villani A, Fabbrocini G, Costa C, Scalvenzi M. Vismodegib treatment in advanced basal cell carcinomas: Real-life experience. Dermatol Ther. 2022;35(1):e15195. 34751490 10.1111/dth.15195 PMC9287051

[20] Gürbüz M, Doğan İ, Akkuş E, Ermiş H, Utkan G, Vatansever S, et al. Efficacy and tolerability of vismodegib treatment in locally advanced and metastatic basal cell carcinoma: retrospective real-life data. Dermatol Ther. 2021;34(6):e15122. 34478210 10.1111/dth.15122

[21] Ruiz-Salas V, Podlipnik S, Sandoval-Clavijo A, Sanmartin-Jiménez O, Bernia-Petit E, Bonfill-Ortí M, et al. Real-World experience with vismodegib on advanced and multiple bccs: data from the RELIVIS study. Dermatology. 2023;239(5):685–93. 37257423 10.1159/000530813

[22] Mannino M, Piccerillo A, Fabbrocini G, Quaglino P, Argenziano G, Dika E, et al. Clinical characteristics of an Italian patient population with advanced BCC and Real-Life evaluation of Hedgehog pathway inhibitor safety and effectiveness. Dermatology. 2023;239(6):868–76. 37311439 10.1159/000531280

[23] Mesti T, Sever M, Ocvirk J. Vismodegib in locally advanced basal cell carcinoma in Slovenia. Dermatology. 2023;239(1):158–64. 35896082 10.1159/000525612 PMC9808722

[24] Gutzmer R, Schulze HJ, Hauschild A, Leiter U, Meier F, Haferkamp S, et al. Effectiveness, safety and utilization of vismodegib in locally advanced basal cell carcinoma under real-world conditions in Germany - The non-interventional study NIELS. J Eur Acad Dermatol Venereol. 2021;35(8):1678–85. 33931910 10.1111/jdv.17332

[25] Apalla Z, Spyridis I, Kyrgidis A, Lazaridou E, Kyriakou A, Fotiadou C, et al. Vismodegib in real-life clinical settings: A multicenter, longitudinal cohort providing long-term data on efficacy and safety. J Am Acad Dermatol. 2021;85(6):1589–92. 33253837 10.1016/j.jaad.2020.11.036

[26] Bendsöe N, Paoli J, Söderkvist K, Persson B, Halldin C, Ihrlund L, et al. A non-interventional study on vismodegib for basal cell carcinoma in Swedish patients. Dermatol Pract Concept. 2023;13(2):e2023211. 10.5826/dpc.1302a211 PMC10188167 37116181

[27] Sekulic A, Yoo S, Kudchadkar R, Guillen J, Rogers G, Chang ALS, et al. Real-world assessment and treatment of locally advanced basal cell carcinoma: findings from the regisonic disease registry. PLoS ONE. 2022;17(1):e0262151. 35030185 10.1371/journal.pone.0262151 PMC8759646

[28] Chang AL, Solomon JA, Hainsworth JD, Goldberg L, McKenna E, Day BM, et al. Expanded access study of patients with advanced basal cell carcinoma treated with the Hedgehog pathway inhibitor, vismodegib. J Am Acad Dermatol. 2014;70(1):60–9. 24189279 10.1016/j.jaad.2013.09.012

[29] Słowińska M, Dudzisz-Śledź M, Sobczuk P, Łasińska I, Pietruszka A, Cybulska-Stopa B, et al. Analysis of efficacy and safety of vismodegib therapy in patients with advanced basal cell carcinoma - real world multicenter cohort study. J Eur Acad Dermatol Venereol. 2022;36(8):1219–28. 35279879 10.1111/jdv.18070 PMC9541446

[30] Herms F, Lambert J, Grob JJ, Haudebourg L, Bagot M, Dalac S, et al. Follow-Up of patients with complete remission of locally advanced basal cell carcinoma after vismodegib discontinuation: A multicenter French study of 116 patients. J Clin Oncol. 2019;37(34):3275–82. 31609670 10.1200/JCO.18.00794

[31] Dréno B, Kunstfeld R, Hauschild A, Fosko S, Zloty D, Labeille B, et al. Two intermittent vismodegib dosing regimens in patients with multiple basal-cell carcinomas (MIKIE): a randomised, regimen-controlled, double-blind, phase 2 trial. Lancet Oncol. 2017;18(3):404–12. 28188086 10.1016/S1470-2045(17)30072-4

[32] Russi EG, Pelissero A, Melano A, Fillini C, Vigna-Taglianti R, Gianello L, et al. Facial basal cell carcinomas in elderly frail patients treated with low total-dose radiotherapy. Anticancer Res. 2015;35(9):4949–53. 26254393

[33] Dika E, Melotti B, Comito F, Tassone D, Baraldi C, Campione E, et al. Neoadjuvant treatment of basosquamous carcinomas with sonidegib: an innovative approach. Exp Dermatol. 2023;32(11):2038–9. 37432021 10.1111/exd.14882

[34] Mohan SV, Chang J, Li S, Henry AS, Wood DJ, Chang AL. Increased risk of cutaneous squamous cell carcinoma after vismodegib therapy for basal cell carcinoma. JAMA Dermatol. 2016;152(5):527–32. 26914338 10.1001/jamadermatol.2015.4330

[35] Bhutani T, Abrouk M, Sima CS, Sadetsky N, Hou J, Caro I, et al. Risk of cutaneous squamous cell carcinoma after treatment of basal cell carcinoma with vismodegib. J Am Acad Dermatol. 2017;77(4):713–8. 28780365 10.1016/j.jaad.2017.03.038

[36] Atwood SX, Chang AL, Oro AE. Hedgehog pathway Inhibition and the race against tumor evolution. J Cell Biol. 2012;199(2):193–7. 23071148 10.1083/jcb.201207140 PMC3471227

[37] Chang AL, Oro AE. Initial assessment of tumor regrowth after vismodegib in advanced basal cell carcinoma. Arch Dermatol. 2012;148(11):1324–5. 22910979 10.1001/archdermatol.2012.2354 PMC3777384

[38] Stratigos AJ, Sekulic A, Peris K, Bechter O, Prey S, Kaatz M, et al. Cemiplimab in locally advanced basal cell carcinoma after Hedgehog inhibitor therapy: an open-label, multi-centre, single-arm, phase 2 trial. Lancet Oncol. 2021;22(6):848–57. 34000246 10.1016/S1470-2045(21)00126-1

[39] Lewis KD, Peris K, Sekulic A, Stratigos AJ, Dunn L, Eroglu Z, et al. Final analysis of phase II results with Cemiplimab in metastatic basal cell carcinoma after Hedgehog pathway inhibitors. Ann Oncol. 2024;35(2):221–8. 38072158 10.1016/j.annonc.2023.10.123

[40] De Giorgi V, Trane L, Savarese I, Silvestri F, Venturi F, Zuccaro B, et al. Lasting response after discontinuation of Cemiplimab in a patient with locally advanced basal cell carcinoma. Clin Exp Dermatol. 2021. 10.1111/ced.14804. 34157152 10.1111/ced.14804

